# Targeting obstetric providers in interventions for obesity and gestational weight gain: A systematic review

**DOI:** 10.1371/journal.pone.0205268

**Published:** 2018-10-05

**Authors:** Michelle A. Kominiarek, Linda C. O’Dwyer, Melissa A. Simon, Beth A. Plunkett

**Affiliations:** 1 Department of Obstetrics and Gynecology, Division of Maternal-Fetal Medicine, Northwestern University, Chicago, Illinois, United States of America; 2 Galter Health Sciences Library, Feinberg School of Medicine, Northwestern University, Chicago, Illinois, United States of America; 3 Department of Obstetrics and Gynecology, Northwestern University, Chicago, Illinois, United States of America; 4 Department of Obstetrics and Gynecology, Division of Maternal-Fetal Medicine, NorthShore University HealthSystem, Evanston, Illinois, United States of America; University of Tennessee Health Science Center, UNITED STATES

## Abstract

**Background:**

Providers need to be comfortable addressing obesity and gestational weight gain so they may give appropriate care; however, health care providers lack guidelines for the most effective educational strategies to assist in providing optimal care.

**Objective:**

To identify studies that involved the obstetric provider in interventions for either the perinatal management of obesity and/or gestational weight gain in a systematic review.

**Search strategy:**

A keyword search of databases was performed up to April 2017.

**Selection criteria:**

Obstetric providers who participated in an intervention with the aim to change a provider’s clinical practice, knowledge, and/or satisfaction with the intervention in relation to the perinatal management of obesity or gestational weight gain were included. Provider intervention could include training or education, changes in systems or organization of care, or resources to support practice. PROSPERO database #42016038921.

**Data collection and analysis:**

Bias was assessed according to the validated Mixed Methods Appraisal Tool. The following variables were synthesized: study location and setting, provider and patient characteristics, intervention features, outcomes and efficacy, and strengths and weakness.

**Main results:**

Of the 6,821 abstracts screened, seven studies (4 quantitative, 3 mixed-methods) with a total of 335 providers met the inclusion criteria; two of which focused on the management of obesity, three focused on gestational weight gain, and two focused on both topics. Interventions that incorporated motivational interviewing skills (n = 2), required additional training for the research study and addressed specific knowledge deficits such as nutrition and exercise (n = 3), and interfaced with the electronic medical record (n = 1) demonstrated the greatest impact on provider outcomes. Provider reported satisfaction scores were generally favorable, but none addressed provider-level efficacy in practice change.

**Conclusions:**

Given the limited number of studies, varying range of provider participation, and lack of provider-level efficacy, further evaluation of provider training and involvement in interventions for perinatal obesity or gestational weight gain is indicated to determine best practices for provider and patient outcomes.

## Introduction

The morbidity associated with obesity is magnified in pregnancy, as the risk for pregnancy-related hypertension and diabetes, cesarean delivery, birth defects, abnormal fetal growth, and stillbirth all increase. [1] Among obese women, a gestational weight gain of 11–20 pounds, as recommended in the 2009 National Academy of Medicine guidelines, can improve short- and long-term maternal and neonatal outcomes; yet, up to 60% of obese women exceed these gestational weight gain guidelines and consequently they are at risk for cesarean deliveries, large-for-gestational-age infants, and postpartum weight retention. [2] Nonetheless, women of all body mass index categories encounter excess morbidity including cesarean deliveries and macrosomia when gestational weight gain is excessive. [3, 4]

Several national organizations recommend that prenatal care providers counsel obese women about the risks of obesity in pregnancy, and incorporate into their care early screening for gestational diabetes, diet and exercise counseling, and anesthesia consults for women with obstructive sleep apnea. [5] The Association of Women’s Health, Obstetric and Neonatal Nurses also recommends to counsel women on the risks of obesity in pregnancy and to provide an interdisciplinary team of experts who promote healthy behaviors during their prenatal care. [6] However, prenatal care providers do not consistently follow these recommendations, especially as it pertains to gestational weight gain counseling. [7–9] In one study of 58 providers from a multi-specialty obstetrical practice in Massachusetts, only 26% of providers routinely ordered glucose tolerance testing during the first trimester, 14% referred patients to a nutritionist, and 3% planned for an anesthesia referral for their obese patients. [10] A cross-sectional survey of 900 members of the American College of Obstetricians and Gynecologists revealed that 42% of obstetrician-gynecologists counseled pregnant women about gestational weight gain “most of the time” and only 36% modified their recommendations for gestational weight gain based on a woman’s pre-pregnancy body mass index. [11]

Moreover, providers may inadvertently contribute to inappropriate gestational weight gain when their advice differs from the recommendations. [7] In focus group studies of 52 obstetric providers from the San Francisco Bay Area, a nurse practitioner intentionally told women to gain more than the recommendations so women didn’t feel as though they failed when they had excessive gestational weight gain. This study also revealed that these providers disagreed about how much to tell women about the risks of obesity in pregnancy. [12] To highlight the role of the provider in gestational weight gain counseling, a systematic review and meta-analysis of health behavior interventions for overweight or obese women found that nutrition and exercise counseling delivered by prenatal care providers (e.g., physicians or midwives) had greater reductions in gestational weight gain compared to interventions delivered by other members of the health care team or research staff. [13] It is essential that providers feel comfortable addressing obesity and gestational weight gain so they may give appropriate care [9]; however, health care providers lack guidelines as to the most effective strategies to assist in providing optimal care. The objective of this study was to perform a systematic review of the literature to identify studies that specifically targeted the obstetric provider in interventions that focused either on the perinatal management of obesity and/or gestational weight gain. The research questions for this systematic review were: (1) Are there education programs or interventions specifically for obstetric providers that focus on the perinatal management of obesity and/or meeting gestational weight gain goals? (2) Can their techniques and modes of intervention be described in detail? (3) If yes to (1) and (2), what is (a) their efficacy in changing obstetric provider behaviors in managing perinatal obesity and/or gestational weight gain or (b) their efficacy in promoting adherence to the gestational weight gain guidelines and other perinatal outcomes.

## Materials and methods

A librarian (L.C.O.) collaboratively developed the search strategies with the other authors (M.A.K., B.A.P.) and ran the searches in the following databases: PubMed MEDLINE, clinicaltrials.gov, Embase (embase.com), Cochrane Central Register of Controlled Trials (CENTRAL) on the Wiley platform, CINAHL (Ebsco), and PsycINFO (Ebsco). The search strategies for the Embase, CENTRAL, CINAHL, and PsycINFO databases were adapted from the MEDLINE search strategy. All databases were searched back to their inception and no language or date limits were applied. Searching for eligible studies to include in the review involved the following approaches: controlled vocabulary (MeSH headings and thesauri of relevant databases) and the keywords of obesity, pregnancy, behavior change, healthcare professionals, trials, and gestational weight gain. Complete search strategies are provided (S1 File). We also attempted to discover additional studies by searching the reference lists of key studies and relevant systematic reviews. The review protocol was registered on December 5, 2016 in the PROSPERO database (#42016038921). The search was completed in April 2017.

The specific inclusion criteria were: (1) Obstetric care providers who participate in at least one intervention, preferably with the provider being the unit of analysis, with the aim (i.e., outcome) to change a providers’ clinical practice (as demonstrated by chart reviews or surveys, for example), knowledge (as demonstrated by pre/post-tests, for example), and/or satisfaction (as demonstrated by surveys) with the intervention in relation to the management of perinatal obesity or gestational weight gain; (2) Type of provider intervention could include training or education interventions, interventions that changed systems or organization of care, provision of resources to support practice (checklists, reminders, etc.); and (3) Randomized controlled trial (RCT), prospective or retrospective cohort, or case-control studies. We specifically excluded interventions that focused only on the patient; however, if the included studies had provider and patient interventions, we reported findings from both of them.

All citations were independently screened by two reviewers (M.A.K., B.A.P.) using the Covidence tool. Once agreement was obtained on articles meeting criteria for full-text review, two reviewers (M.A.K., B.A.P.) independently extracted the following data from each article: (1) study location and setting, (2) provider characteristics (number, provider type, and demographics, etc.); (3) intervention features (type, duration, frequency, etc.); (4) outcomes and efficacy; (5) study design; and (6) strengths and weakness of each study. These data were summarized for each full-text article. Where applicable, differences in means or proportions from pre- and post-testing were reported.

Due to the combination of quantitative, qualitative, and mixed-methods studies that met criteria for review including RCTs, non-RCTs, and descriptive studies, the risk of bias was evaluated according to the validated Mixed Methods Appraisal Tool. [14] This tool initially proposes two screening questions to assess whether there is an appropriate research objective and whether the research design allows this objective to be addressed. Studies are then rated based on four areas of methodology, the specifics of which vary depending on study methodology. Studies were assessed as either meeting the criteria, or not, and each area represents 25% of the total quality score (ranging from 0% to 100%). If studies had both quantitative and qualitative components, they were assessed separately and also as a mixed-method study. The total score could not exceed the lowest score of the individual study components. Both M.A.K. and B.A.P. independently assessed bias and then came to agreement on scores whereby an overall quality score was calculated with ranges from 25% to 100% for each of the studies. Bias was assessed according to the unit of analysis for each study (e.g., provider, patients, or both). This systematic review was determined to be exempt from the Northwestern University IRB given that it was a review of previously published studies. This work was supported by Grant Number K23HD076010 from the Eunice Kennedy Shriver National Institute of Child Health & Human Development of the National Institutes of Health.(M.A.K) The funding source had no role in the study design; the collection, analysis and interpretation of data; in the writing of the report; and in the decision to submit the article for publication.

## Results

**Fig 1:**
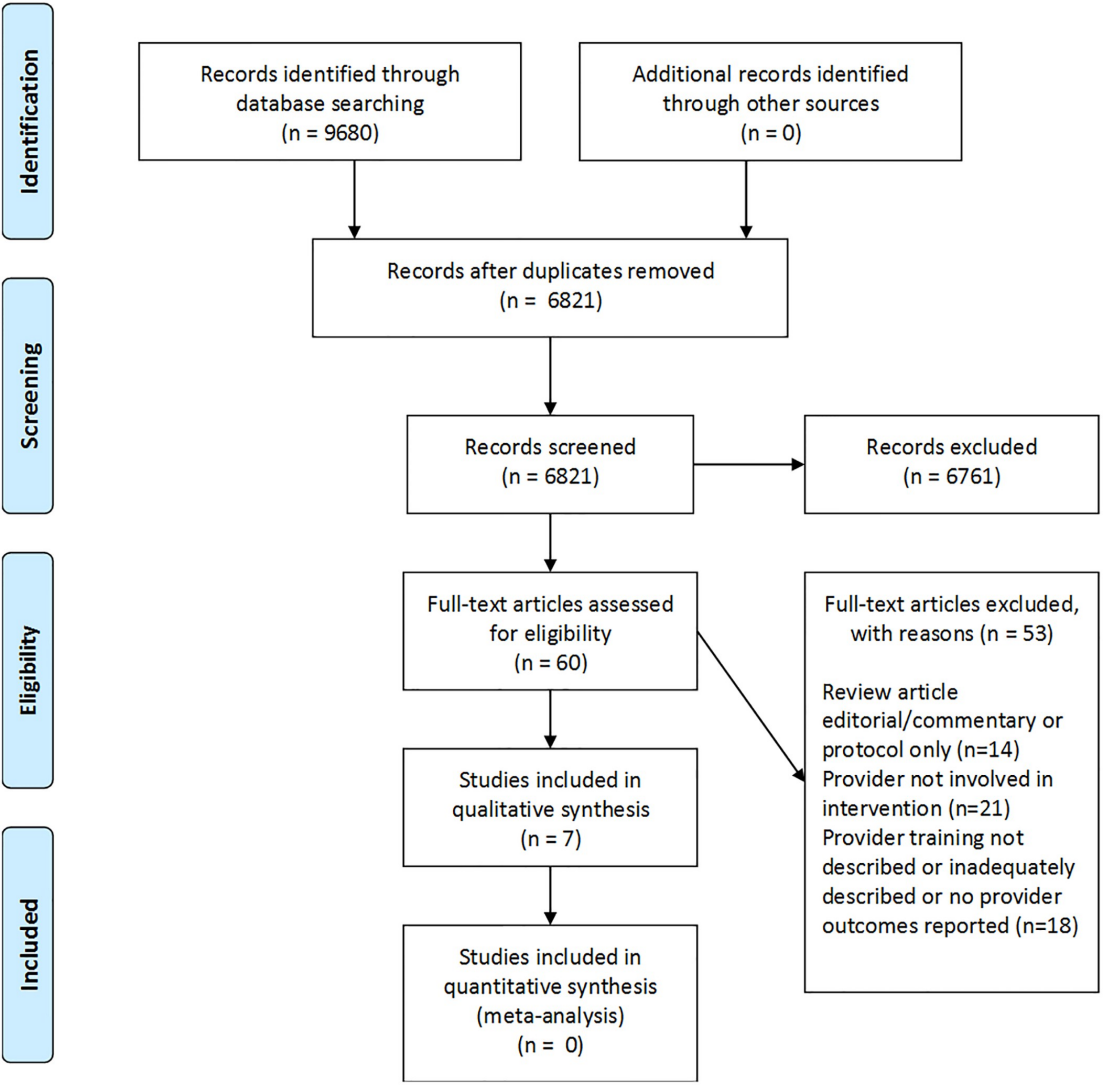
Flowchart of study selection.

Of the 6821 abstracts screened, 60 full-text studies were reviewed after conflict resolution by M.A.K. and B.A.P.(Fig 1) The most common reasons for exclusion of studies at the initial phase (6821 abstracts) were intervention not related to pregnancy, intervention focused on post-partum period only, and intervention focused on patients only. A total of seven studies met the inclusion and exclusion criteria for this study. These studies are summarized in Table 1. Of the seven studies, two focused on the management of obesity, three focused on gestational weight gain, and two focused on both topics.

**Table 1:** Description of studies included in the systematic review.

Author/Year	Study Design/Year	Provider Characteristics	Patient Characteristics	Country/Setting	Inclusion/Exclusion for providers	Intervention	Provider Outcomes	Unit of analysis	Limitations
Lindberg 2014 [15]	Retrospective cohort of pregnant women 2011–2012 with pre vs. post analytical design	Obstetricians, family physicians, or nurse midwives	83–78% white Normal pre-pregnancy BMI 44–48% nulliparas	United States Multi-site health care system in the Midwest at 5 ambulatory clinics	No specific exclusion criteria for providers	(1) Pilot program for GWG counseling with “best practice alerts” using EMR smart sets (2) Didactic Grand Rounds presentation	2009 NAM consistent GWG counseling improved from 2.6% (n = 388 charts) to 51.0% (n = 345 charts), p<0.001	Patient EMR	Total number of providers and their demographics not known Lack of provider-level outcomes
Lindhardt 2014 [16]	Prospective cohort with pre vs. post- analytical design Dates not specified	12 women with 4–30 years’ work experience as HCP	Not applicable	Southern Denmark region	Able to be released from work duties to attend training	3 day training course in MI for women with obesity	Overall MI scores increased in the “majority” but no change in empathy scores based on review of audiotaped consultations in 11/12 participants	HCP	Selection bias for HCP Lack of data and statistical analysis for the MI scores
Kinnunen 2008 [17]	Non-randomized controlled trial of pregnant women	23 PHN at six maternity and child health clinics 14 PHN in intervention, 9 PHN in control clinics 71% ≥ 40 years old 3.5 years median time working in maternity clinic	77% participation rate 15% drop-out rate Data collection in >96% of fields 87% completed weekly diet and exercise logs	City of Tampere, Town of Hameenlinna, Finland Maternity and child health clinics in primary care	No specific exclusion criteria for providers	Patient counseling on physical activity, diet and preventing excessive GWG at sessions using “counseling cards” with 6 and 12 hours of training for control and intervention PHN, respectively	Mean satisfaction scores: 3.4 ± 1.2 study arrangements, 3.9 ± 1.1 physical activity counseling 3.6 ± 1.1 dietary counseling for intervention PHN; 3.9 ± 0.7 study arrangements for control PHN “Nearly all [PHN] regarded training and support as sufficient.”	PHN for experience assessment	Only satisfaction and not specific knowledge change assessed Lack of provider-level data
Daley 2015 [18]	(1)Randomized controlled trial of pregnant women (2) Qualitative analysis of participating midwives 2012–2013	7 of 8 community midwives completed qualitative study 6–29 years’ experience	36 low-risk women in usual care vs. 40 in intervention, 28 years old, 85–91% white ethnicity, 28–62% nulliparous, BMI 18–29.9kg/m^2^ at 1st appointment	United Kingdom	No specific exclusion criteria for providers	Attended 60–70 minute training course Midwives weighed and plotted weight on a chart, setting GWG goals, giving brief feedback at each appointment, encouraging women to weigh themselves between appointments	(1) Quantitative: 35/37 charts reviewed had GWG data completed with at least 80% accuracy (2) Qualitative: No difficulty in discussing GWG or regular weighing Feasible to deliver in 1–3 minutes and incorporate into a 10-15 min time slot	(1) Patient EMR for quantitative (2) Midwives for qualitative	Relevance restricted to where weighing is not routine in pregnancy Limited provider-level data Lack of patient demographic data
Davis 2012 [19]	Mixed-methods program evaluation of a service development initiative for overweight or obese women to limit GWG 4/2010-12/2010	Midwives responsible for offering service	BMI > 25 kg/m^2^	South East Sydney and Central Coast of New South Wales, community settings	No specific exclusion criteria for providers	Training in nutrition, exercise, group facilitation skills, breastfeeding challenges, risks of obesity, and how to talk to women about their weight Included expert in MI	Qualitative from 4 focus groups: Difficulty finding the words (not wanting to offend), acknowledging challenges (transportation, childcare, time), midwife’s knowledge (varied, some wanted more education) Raised awareness of obesity Made more referrals to dieticians Changed their practice and discussed weight for all patients Gained more confidence Quantitative: 81/232 women agreed to participate; women with BMI>35 kg/m^2^ twice as likely to accept service; most common reason for declining was scheduling conflicts	(1) Midwives (2) Patient	Length of training and number of midwives implementing service unknown Lack of patient and provider-level demographics
Basu 2014 [20]	Prospective cohort with pre vs. post-analytic design 2013	32 of 43 registered midwives attended sessions including Community and integrated midwives at NHS pay bands 6 and 7	Not available	Wales, UK Single Health Board accounting for 20.2% of deliveries in 40 training places accounting for 41% of the midwifery workforce	Community midwives employed within Health Board	4 Training sessions over 3.5 hours on MI, nutrition, physical activity, weight management delivered by RD with group discussions and short lectures Learning objectives to understand risks of obesity in pregnancy, nutritional requirements, recommendations for physical activity, how to support women to maintain healthy weight	Improved knowledge and confidence (p<0.005) per self-report Better knowledge of pregnancy-specific food and nutrition, GWG goals, confidence to explain risks of obesity 100% rated program as “excellent” or “good”	Midwives	Actual knowledge and change in practice not reported Lack of provider and patient demographic data
Heslehurst 2015 [21]	Simultaneous mixed methods study with (1) provider surveys and (2) case audits of a maternal obesity care pathway	243 healthcare professionals (community based midwives, consultant obstetricians, consultant anesthetists, trainee obstetricians	Charts of 60 women with booking BMI > 40 kg/m^2^	Multidisciplinary group in a large NHS Trust in Northeast England	No specific exclusion criteria for providers	No specific training sessions described	(1) Several communication barriers, wanted more training based on responses to surveys (2) 75% mean antenatal compliance with pathways for 27 records	(1) HCP (2) Patient medical record	No provider level compliance with pathways or practice changes Lack of provider and patient demographic data

HCP Health Care Professional

PHN Public Health Nurse

RD registered dietician

NHS National Health Service

MI Motivational interviewing

GWG gestational weight gain

NAM National Academy of Medicine

EMR electronic medical record

The overall quality scores of the seven studies ranged from 25% to 100%, with a median value of 50% (Table 2) and a wide distribution of scores over the quantitative (n = 4) and mixed-methods (n = 3) studies. Of the six studies that had a quantitative non-RCT component, three had selection bias for the providers in that they may have opted to participate in the intervention due to convenience [16, 17, 20] and two did not account for baseline differences between comparison groups. [17, 19] Of the three studies that had a qualitative component, two did not account for the study context in the findings (e.g., would the findings be similar in different settings) or how the researchers themselves may have influenced the findings. [18, 19] The three highest scoring studies (≥75%) are described in greater detail below. [15, 16, 21]

**Table 2:** Bias assessment with the Mixed Methods Appraisal Tool.

Types of mixed methods Study components	Methodological quality criteria	Lindberg 2014 [15] Quantitative	Lindhardt 2014 [16] Quantitative	Kinnunen 2008 [17] Quantitative	Daley 2015 [18] RCT + Qualitative	Davis 2012 [19] Quantitative + Qualitative	Basu 2014 [20] Quantitative	Heslehurst 2015 [21] [21] [21] Quantitative + Qualitative
**Screening questions**	Are there clear qualitative and quantitative research questions (or objectives), or a clear mixed methods question (or objective)?	**Yes**	**Yes**	**Yes**	**Yes**	**Yes**	**Yes**	**Yes**
	Do the collected data address the research question (objective)?	Yes	Yes	Yes	Yes	Yes	Yes	Yes
**1. Qualitative**	1.1. Are the sources of qualitative data (archives, documents, informants, observations) relevant to address the research question (objective)?				**Yes**	**Yes**		**Yes**
	1.2. Is the process for analyzing qualitative data relevant to address the research question (objective)?				**Yes**	**Yes**		**Yes**
	1.3. Is appropriate consideration given to how findings relate to the context, e.g., the setting, in which the data were collected?				**No**	**No**		**Yes**
	1.4. Is appropriate consideration given to how findings relate to researchers’ influence, e.g., through their interactions with participants?				**No**	**No**		**Yes**
**2. Quantitative RCT**	2.1. Is there a clear description of the randomization (or an appropriate sequence generation)?				**Yes**			
	2.2. Is there a clear description of the allocation concealment (or blinding when applicable)?				**Yes**			
	2.3. Are there complete outcome data (≥80%)?				**Yes**			
	2.4. Is there low withdrawal/drop-out (<20%)?				**Yes**			
**3. Quantitative non-RCT**	3.1. Are participants (organizations) recruited in a way that minimizes selection bias?	**Yes**	**No**	**No**		**Yes**	**No**	**Yes**
	3.2. Are measurements appropriate (clear origin, or validity known, or standard instrument; and absence of contamination between groups when appropriate) regarding the exposure/intervention and outcomes?	**Yes**	**Yes**	**Yes**		**Yes**	**No**	**Yes**
	3.3. In the groups being compared (exposed vs. non-exposed; with intervention vs. without; cases vs. controls), are the participants comparable, or do researchers take into account (control for) the difference between these groups?	**Yes**	**Yes**	**No**		**No**	**Yes**	**Yes**
	3.4. Are there complete outcome data (80% or above), and, when applicable, an acceptable response rate (60% or above), or an acceptable follow-up rate for cohort studies (depending on the duration of follow-up)?	**Yes**	**Yes**	**No**		**Yes**	**Yes**	**Yes**
**5. Mixed methods**	5.1. Is the mixed methods research design relevant to address the qualitative and quantitative research questions (or objectives), or the qualitative and quantitative aspects of the mixed methods question (or objective)?				**Yes**	**Yes**		**Yes**
	5.2. Is the integration of qualitative and quantitative data (or results*) relevant to address the research question (objective)?				**Yes**	**Yes**		**Yes**
	5.3. Is appropriate consideration given to the limitations associated with this integration, e.g., the divergence of qualitative and quantitative data (or results*) in a triangulation design?				**No**	**No**		**Yes**
**Total**		**100% (4/4)**	**75% (3/4)**	**25% (1/4)**	**50% (2/4)**	**50% (2/4)**	**50% (2/4)**	**100%(8/8)**

RCT Randomized controlled trial

Lindberg *et al* developed an intervention that entailed a “best practice alert” embedded into the EMR of obstetric patients during 2011–2012 at a multi-site health care system in the United States. [15] A group of providers (total number not specified) piloted the program, provided feedback and designed the intervention to include the following elements: automatic calculation of pre-pregnancy body mass index, individualized calculation of gestational weight gain, individualized gestational weight gain recommendations as promoted by the National Academy of Medicine, and a template for scripted counseling, documentation, and personalized recommendations for the patient. Prior to the roll-out of the program, nursing protocols were developed and provider education was provided through a didactic Grand Rounds session which focused on the topic of gestational weight gain and introduced the new best practice alert to providers. Overall, there was a change in the pattern of gestational weight gain counseling such that counseling consistent with the 2009 National Academy of Medicine guidelines increased from 2.6% before to 51.0% after the program (p<0.001) per a review of the electronic medical records. Counseling consistent with the 2009 National Academy of Medicine guidelines improved among all provider types (3.0% vs. 55.9% for obstetricians, 1.8% vs. 37.3% for family physicians, and 1.4% vs. 38.5% for nurse midwives, p<0.001 for all comparisons). The proportion of women with a documented pre-pregnancy body mass index increased from 79.9% to 91.3%, p<0.001.

In 2014, Lindhart *et al* aimed to determine if 12 health care professionals in Denmark could improve their motivational interviewing skills when communicating with obese pregnant women after attending a 3-day training course in motivational interviewing. [16] Two certified members of the Motivational Interviewing Network Trainers facilitated the training and focused on the four principles of motivational interviewing (expressions of empathy, development of discrepancy, rolling with resistance, supporting the individuals’ self-efficacy) and five specific counseling techniques (open-ended questions, reflective listening, affirmations, summarizing, eliciting change talk). Training also included instruction, role-playing, and theoretical lectures in addition to supervised individual and group work. The primary outcome of the study was achievement of motivational interviewing skills as determined by a review of audio files. Global rating and global spirit scores increased in the “majority” of health care professionals and decreased in none, but actual scores and statistical test results were not provided. The behavior frequencies changed in a direction that was appropriate to motivational interviewing for open questions, total questions, and motivational interviewing adherent behaviors; however, there was an increase in closed questions and giving information and a decrease in simple reflections according to the median values of the total score. One health care professional did not achieve proficiency and 3 achieved competency on the global rating. Of interest, there was no difference in empathy scores before and after training, one of the key components of motivational interviewing.

In 2015, Heselhurst *et al* evaluated the implementation of maternal obesity care pathways or management algorithms of the National Health Service in the United Kingdom. The evaluation utilized a mixed-methods approach and considered the perspective of multiple stakeholders (medical records of 27 obese pregnant women, 243 prenatal care providers) and used quantitative and qualitative postal surveys and case audits of compliance in delivering the pathways. [21] The content of these pathways included the best available published evidence and were subjected to an iterative process of updating according to new recommendations or evidence, but how the provider was trained in implementing the pathways was not described. For the current systematic review, we focused on the evaluation pertaining to the providers. According to the postal surveys, 90% of provider participants were aware of the care pathways and felt they were worthwhile, facilitated good practice, and increased confidence, but more training regarding the pathways and clinical care (e.g., weight gain advice, safety of exercise, how to approach conversations with sensitivity) was requested. The qualitative data identified several barriers to communication which included stigma that may be associated with obesity, making patients feel uncomfortable, providers feeling uncomfortable with their own weight, providers feeling judgmental or overly negative, and providers feeling they are limiting women’s choices.

The case audits were limited to women with a booking body mass index >40 kg/m^2^ whereby the hand-held antenatal records for randomly selected women were reviewed and assessed for compliance with elements of the antenatal care pathway. Of the 27 records reviewed, antenatal mean compliance across all areas assessed was 75%. Areas that scored below the 75% cut-off included offering vitamin D supplements, using appropriate-sized blood pressure cuff, weighing at 28 and 32 weeks, anesthesia consult, and reinforcement of diet and physical activity goals. The strengths of this study include its multi-dimensional evaluation process from the perspective of a variety of stakeholders. Objective data from the chart audit provides a measure of efficacy of the intervention pathway, but they were not specific to the provider.

We summarize the findings from the seven studies as follows (Table 1): The studies varied from whether they focused only on the prenatal care provider [16, 17, 20] vs. a combined patient-provider intervention. [15, 18, 19, 21] There was also a range in study designs from small feasibility studies occurring in the context of original research [15, 16, 18, 20] to evaluations of local or national service programs. [17, 19, 21] Overall, the provider sample size was small with a total of 335 among 6 studies with a range of 8–243 providers per study. Lindberg et al did not specify the provider sample size. [15] Provider-level efficacy was also lacking in all of these studies. For example, providers may have improved some of their skills in motivational interviewing or increased knowledge [16], but how the intervention influenced their actual practice was not studied. Individual provider chart review would have provided these data; however, the studies assessed patient-level data only through compliance audits. [15, 18, 21] Five of the studies evaluated provider satisfaction with the training program with either qualitative or quantitative methods. [17–21] For example, in Kinnunen et al’s study, mean satisfaction scores for the providers who received a 12 hour training session prior to implementation of the health behavior intervention for their patients ranged from 3.4–3.9 out of 5 points and “nearly all regarded the training as important”, but implementing the study took too much time, estimated at 40–60 min/visit in the intervention and 10–20 min/visit in the control clinics. [17] The evaluations of the other interventions from Basu et al, Davis et al, and Daley et al were mostly positive containing comments such as “feasible to deliver”, “changed practice”, “gained more confidence”, and “improved knowledge”. [18–21]

We noted several other similarities among the seven studies in that the majority of provider participants were midwives whose practice was based in the United Kingdom (n = 3), Denmark, Finland, Australia, and the United States. Practices such as routine weighing are common in some countries such as the United States, whereas the American College of Obstetricians and Gynecologists’ (ACOG) guidelines do not advise routine vitamin D supplementation in obese women. [21] Motivational interviewing themes or skills were common components of the provider education and training. Lindhart et al objectively evaluated the efficacy of motivational interviewing training in a pre-post study design [16], whereas two other studies incorporated motivational interviewing in the training, but did not evaluate provider change in these skills. [19, 20] Other common themes among the studies were provider lack of knowledge and confidence in either the management of perinatal obesity or gestational weight gain and communication issues (e.g., providers couldn’t “find the words” to discuss these sensitive topics). [19–21] Lastly, none of the seven studies reported information on provider or patient race, ethnicity, socioeconomic status, or other cultural influences that may significantly impact approaches to weight management.

## Discussion

### Main findings

In this systematic review of provider involvement in either maternal obesity or gestational weight gain interventions, we found only seven articles that met our inclusion and exclusion criteria among 6,821 abstracts. There is a dearth of information on provider involvement in such interventions, either as a primary provider or as a member of a multi-disciplinary team in the training and management of these important maternal health issues. As determined from this systematic review, there are many intervention components that have the potential to change provider behavior including interfaces with the EMR, training in motivational interviewing and implementation of maternal care pathways or treatment algorithms. Our study adds to the literature in that it has now identified seven studies that have incorporated the obstetric provider into an intervention or specifically targeted obstetric providers in an intervention with provider-level data as part of a program evaluation; however, the quality of the evidence to support these recommendations was low given that the median bias assessment score was 50%.

### Strengths and limitations

This systematic review provides a comprehensive and updated assessment of the literature and used a validated quality assessment tool for mixed-methods studies. Future research can adapt these studies, evaluate provider involvement in a more rigorous fashion, and further strengthen the evidence base. We recognize some limitations as a result of the quality of the individual studies including small sample size, convenience sampling, and no universal approach to weight management (e.g., sites where weighing pregnant women is not routine). Due to the heterogeneity of the individual components of the provider interventions in this systematic review (i.e., motivational interviewing course, didactic sessions to increase knowledge, etc.) and the varying approaches to data reporting and analysis, we were not able to perform a meta-analysis to further evaluate their efficacy. Furthermore, none of the studies evaluated the efficacy and sustainability of the intervention on a provider-level basis in a given clinical practice. We acknowledge it is difficult to report individual provider outcomes such as change in knowledge or EMR documentation and maintain provider confidentiality in studies with a small provider sample size.

Of all the behavior changes that need to occur to effect improvement in health, we realize that the provider is just one part of the entire team that includes patients, other healthcare professionals, healthcare systems, and national organizations. Even if providers can improve knowledge and demonstrate behavior change with respect to their clinical practice, ultimately the primary question is whether there is a cumulative positive effect on patient outcomes such as improving their health behaviors or meeting gestational weight gain goals. For example, Kinnunen et al concluded from their pilot study, which required 12 hours of training for PHNs in the intervention group, that the strategy was feasible. [17] This study then served as the basis for a larger cluster-RCT that utilized a similar intervention strategy. [17] Ultimately, their cluster RCT did not show differences between gestational weight gain and other outcomes such as gestational diabetes between the intervention and control clinics, but there was a reduction in birth weight for women in the intervention arm. [22] The subsequent cluster-RCT did not include provider outcomes, so it was not included in this systematic review. Further evaluation of provider training and involvement is indicated to determine best practices for provider and patient outcomes.

### Interpretation

Other studies that target either perinatal obesity or gestational weight gain in health behavior interventions for patients typically use nutritionists, physical therapists, psychologists, and other research personnel, but not the actual prenatal care provider to deliver the intervention. Indeed, a systematic review of nine electronic databases in 2014 documented that none of the 3,608 identified studies in which interventions were used to improve gestational weight gain explicitly targeted obstetric providers in their intervention. [23] In 2011, another systematic review of health behavior interventions for weight management in pregnancy acknowledged that obstetric providers were not trained in any of the studied interventions and called for their inclusion in future studies. [24] It is clear based on this systematic review and other published studies that providers, including physicians and midwives, have not received sufficient training to knowledgably discuss weight and healthy behaviors. [11, 12, 24] Curriculum and competencies vary between physicians and nurses, though nurses and nurse midwives typically report greater comfort and knowledge in discussing topics related to weight and nutrition than physicians which may result from a more structured curriculum offered during their training. [25] Other studies have suggested the types of training that would be more likely to improve provider knowledge and the quality of counseling with respect to perinatal obesity and gestational weight gain. [10, 26–29] Components identified as most effective in changing provider behavior are assessment of learning needs, sequenced and multifaceted educational activities, and opportunities to practice learned skills. [30] Thus, changes in practice are best engendered by more than didactic sessions alone, but through the use of interactive techniques (e.g., reminders, simulations). [31–34] Furthermore, audit and feedback have been shown to be useful in causing physicians to change their behaviors. [35] Motivational interviewing, a goal-oriented, individual-centered counseling style for eliciting behavior change and often cited as an intervention component for weight-related issues, typically is not part of medical training. Motivational interviewing has been recommended to be a component of continuing medical education activities since it has also has proven effective in eliciting behavior change that contributes to improved health outcomes and patient-provider communication. [36, 37]

There are likely other issues to consider for provider interventions such as a perception that counseling about weight-related issues is futile or not important, [12, 28, 34] providers’ conflicted feelings about their own body weight and image, and how race, ethnicity and culture influence diet and exercise during pregnancy. [38] Certainly time constraints of routine prenatal visits make counseling about weight and related health behaviors difficult. [7, 39]

## Conclusions

Obesity and gestational weight gain are serious problems in pregnancy. Not only are there short-term implications for perinatal outcomes, but long-term maternal and child health are at risk too. [2] If providers can enhance their knowledge of obesity risks and skills in gestational weight gain counseling, it is likely that they can better assist women in meeting their gestational weight gain goals and perhaps influence other outcomes. Thus, obstetric providers should be participants in trials that assess the management of perinatal obesity and health behavior interventions for improving gestational weight gain and related perinatal outcomes so that their involvement can be more formally evaluated with respect to patient outcomes.

## Supporting information

S1 File Complete search strategies for the systematic review. (DOCX)

S2 File PRISMA 2009 checklist. (PDF)
