# Supplementary material for: Targeting obstetric providers in interventions for obesity and gestational weight gain: A systematic review
Source: PLoS One. 2018 Oct 5;13(10):e0205268. doi: 10.1371/journal.pone.0205268 (PMC6173456; doi:10.1371/journal.pone.0205268)
Supplement: S1 File — (DOCX) [file pone.0205268.s001.docx]

**Search strings appendix: Kominiarek SR**

**Search terms in each database were exploded (i.e. searches included possibly relevant terms below the primary term in the search terms thesauri). We also performed title/abstract searches to ensure maximum retrieval.**

**PubMed MEDLINE**

("Pregnancy"[Mesh] OR "Prenatal Care"[Mesh] OR "Pregnancy Complications"[Mesh] OR "Prenatal Injuries"[Mesh] OR "Prenatal Diagnosis"[Mesh] OR “Pregnancy”[tiab] OR “Pregnancies”[tiab] OR “Pregnant”[tiab] OR “Gestation” OR “Prenatal” OR “Pre-natal” OR “Antenatal”[tiab])

AND

("Obesity"[Mesh] OR "Weight Gain"[Mesh] OR “obesity”[tiab] OR “obese”[tiab] OR “overweight”[tiab] OR “weight gain”[tiab])

AND

("Prenatal Education"[Mesh] OR "Education"[Mesh] OR "education" [Subheading] OR "Behavior Therapy"[Mesh] OR "Counseling"[Mesh] OR "Health Promotion"[Mesh] OR "Health Behavior"[Mesh] OR "Risk Reduction Behavior"[Mesh] OR "Health Knowledge, Attitudes, Practice"[Mesh] OR “education”[tiab] OR “behavior therapy”[tiab] OR “behavior therapy”[tiab] OR “behavioral therapy”[tiab] OR “behavioural therapy”[tiab] OR “behavior change”[tiab] OR “behavior change”[tiab] OR “behavioral change”[tiab] OR “behavioural change”[tiab] OR “counseling”[tiab] OR “regular weighing”[tiab] OR “training”[tiab] OR “health promotion”[tiab] OR “motivational interviewing”[tiab] OR “counseling”[tiab] OR “Provider knowledge”[tiab] OR “Provider attitudes”[tiab] OR “practice change”[tiab])

**Embase**

'pregnancy'/exp OR 'pregnancy complication'/exp OR ‘high risk pregnancy’/exp OR ‘prenatal care’/exp OR ‘prenatal diagnosis’/exp OR ‘prenatal injury’/exp OR ‘Pregnancy’ OR ‘Pregnancies’ OR ‘Pregnant’ OR ‘Pregnancies’ OR ‘Gestation’ OR ‘Pregnancies’ OR ‘Gestational’ OR ‘Pregnancies’ OR ‘Prenatal’ OR ‘Pregnancies’ OR ‘Pre-natal’ OR ‘Pregnancies’ OR ‘Antenatal’

AND

‘Obesity’/exp OR ‘Weight Gain’/exp OR ‘obesity’ OR ‘obese’ OR ‘overweight’ OR ‘weight gain’

AND

‘childbirth education’/exp OR ‘health Education’/exp OR ‘Behavior Therapy’/exp OR ‘Counseling’/exp OR ‘Health Promotion’/exp OR ‘Health Behavior’/exp OR ‘Risk Reduction’/exp OR ‘attitude to health’/exp OR ‘behavior therapy’ OR ‘behaviour therapy’ OR ‘behavioral therapy’ OR ‘behavioural therapy’ OR ‘behavior change’ OR ‘behavior change’ OR ‘behavioral change’ OR ‘behavioural change’ OR ‘counseling’ OR ‘regular weighing’ OR ‘training’ OR ‘health promotion’ OR ‘motivational interviewing’ OR ‘Provider knowledge’ OR ‘Provider attitudes’ OR ‘practice change’ OR ‘health attitude’ OR ‘health attitudes’

**CINAHL**

(MH "Pregnancy+") OR (MH "Pregnancy, Multiple+") OR (MH "Pregnancy Trimesters+") OR (MH "Pregnancy Complications+") OR (MH "Prenatal Care") OR (MH "Prenatal Diagnosis") OR "prenatal injury" OR ‘Pregnancy’ OR ‘Pregnancies’ OR ‘Pregnant’ OR ‘Pregnancies’ OR ‘Gestation’ OR ‘Pregnancies’ OR ‘Gestational’ OR ‘Pregnancies’ OR ‘Prenatal’ OR ‘Pregnancies’ OR ‘Pre-natal’ OR ‘Pregnancies’ OR ‘Antenatal’

AND

(MH "Obesity+") OR (MH "Weight Gain") OR ‘obesity’ OR ‘obese’ OR ‘overweight’ OR ‘weight gain’

AND

(MH "Childbirth Education") OR (MH "Health Education") OR (MH "Behavior Therapy") OR (MH "Behavior Modification") OR (MH "Counseling") OR (MH "Health Behavior") OR "risk reduction" OR (MH "Attitude to Health") OR (MH "Health Promotion") OR “risk reduction” OR ‘behavioral therapy’ OR ‘behavioural therapy’ OR ‘behavior change’ OR ‘behavior change’ OR ‘behavioral change’ OR ‘behavioural change’ OR ‘counseling’ OR ‘regular weighing’ OR ‘training’ OR ‘health promotion’ OR ‘motivational interviewing’ OR ‘Provider knowledge’ OR ‘Provider attitudes’ OR ‘practice change’ OR ‘health attitude’ OR ‘health attitudes’

**PsycINFO**

(DE "Pregnancy+") OR (DE "Prenatal Care" OR DE "Prenatal Diagnosis") OR "prenatal injury" OR ‘Pregnancy’ OR ‘Pregnancies’ OR ‘Pregnant’ OR ‘Pregnancies’ OR ‘Gestation’ OR ‘Pregnancies’ OR ‘Gestational’ OR ‘Pregnancies’ OR ‘Prenatal’ OR ‘Pregnancies’ OR ‘Pre-natal’ OR ‘Pregnancies’ OR ‘Antenatal’

AND

(DE "Overweight" OR DE "Obesity") OR (DE "Weight Gain") OR ‘obesity’ OR ‘obese’ OR ‘overweight’ OR ‘weight gain’

AND

((((((DE "Health Education" OR DE "Health Promotion") OR (DE "Behavior Therapy" OR DE "Aversion Therapy" OR DE "Conversion Therapy" OR DE "Dialectical Behavior Therapy" OR DE "Exposure Therapy" OR DE "Implosive Therapy" OR DE "Reciprocal Inhibition Therapy" OR DE "Response Cost" OR DE "Systematic Desensitization Therapy")) OR (DE "Behavior Modification")) OR (DE "Counseling")) OR (DE "Health Behavior")) OR (DE "Harm Reduction")) OR (DE "Health Attitudes") OR “risk reduction” OR ‘behavioral therapy’ OR ‘behavioural therapy’ OR ‘behavior change’ OR ‘behavior change’ OR ‘behavioral change’ OR ‘behavioural change’ OR ‘counseling’ OR ‘regular weighing’ OR ‘training’ OR ‘health promotion’ OR ‘motivational interviewing’ OR ‘Provider knowledge’ OR ‘Provider attitudes’ OR ‘practice change’ OR ‘health attitude’ OR ‘health attitudes’

**Cochrane CENTRAL Register of Controlled Trials + Cochrane Database of Systematic reviews**

("Pregnancy" OR “Pregnancies” OR “Pregnant” OR “Gestation” OR “Gestational” OR “Prenatal” OR “Pre-natal” OR “Antenatal”)

AND

("Overweight" OR "Obesity" OR "Weight Gain" OR “obese”)

AND

("Health Education" OR "Health Promotion" OR "Behavior Therapy" OR "Aversion Therapy" OR "Conversion Therapy" OR "Dialectical Behavior Therapy" OR "Exposure Therapy" OR "Implosive Therapy" OR "Reciprocal Inhibition Therapy" OR "Response Cost" OR "Systematic Desensitization Therapy" OR "Behavior Modification" OR "Counseling" OR "Health Behavior" OR "Harm Reduction" OR "Health Attitudes" OR “risk reduction” OR “behavioral therapy” OR “behavioural therapy” OR “behavior change” OR “behavioral change” OR “behavioural change” OR “regular weighing” OR “training” OR “motivational interviewing” OR “Provider knowledge” OR “Provider attitudes” OR “practice change” OR “health attitude” OR “health attitudes”)

_______________________

**ClinicalTrials.gov**

(pregnancy OR pregnant OR pregnancies OR prenatal OR pre-natal OR antenatal OR gestation OR gestational)

AND

(Overweight OR Obesity OR “Weight Gain” OR obese)

AND

("Health Education" OR "Health Promotion" OR "Behavior Therapy" OR "Aversion Therapy" OR "Conversion Therapy" OR "Dialectical Behavior Therapy" OR "Exposure Therapy" OR "Implosive Therapy" OR "Reciprocal Inhibition Therapy" OR "Response Cost" OR "Systematic Desensitization Therapy" OR "Behavior Modification" OR "Counseling" OR "Health Behavior" OR "Harm Reduction" OR "Health Attitudes" OR “risk reduction” OR “behavioral therapy” OR “behavioural therapy” OR “behavior change” OR “behavioral change” OR “behavioural change” OR “regular weighing” OR “training” OR “motivational interviewing” OR “Provider knowledge” OR “Provider attitudes” OR “practice change” OR “health attitude” OR “health attitudes”)
